# Inversion of the Uterus

**Published:** 1858-10

**Authors:** 


### INVERSION OF THE UTERUS.

A case of this kind is reported in the present number of the Journal, which possesses some features of interest. Spontaneous inversion of the uterus, any considerable time after delivery, is a very rare occurrence. Yet if, as Dr. Fisher states, he felt the uterine tumor above the pubes after the placenta was removed, and there were no severe symptoms, such as dangerous flooding, syncope or prostration closely following the expulsion of the child and placenta (or for several weeks afterwards), it is scarcely possible that the inversion took place at the time of delivery. It is proper to state in this connection, that when called to see the patient in consultation with the attending physician, I questioned both the patient and her mother, carefully and minutely, in regard to what transpired at the time of the confinement, and I elicited no facts differing from the statements given by Dr. Fisher. Relying on the correctness of the history given both by the attending physician and the patient, the most rational mode of explaining the occurrence of the inversion, was, by supposing that the shortness of the umbilical cord produced, at the time the child was expelled, sufficient traction to indent the fundus of the womb at the same time that the placenta was rapidly detached. This simple indentation or partial inversion remained without increase so long as the patient remained steadily in the recumbent posture, and she slowly recovered. But so soon as she began to sit in the chair, and the weight of the abdominal viscera was allowed to bear upon the contents of the pelvis, it tended directly to increase the depression of the fundus; and when she added to an erect position of the body beside her piano the act of singing loud and clear, the downward pressure was sufficient to complete the inversion. This explanation is rendered the more probable, from the fact, that the hemorrhage, which ultimately led to the discovery of the inversion, commenced very soon, if not immediately, after the act of singing. In all ordinary cases it is true that the mouth of the uterus becomes so firmly contracted in a few days that a complete inversion could not easily occur. That such contraction does not always take place, however, is fully proved by well authenticated cases. Thus Ana and Baudeloque relate a case of complete inversion that occurred *twelve* days after confinement, from simply straining at stool. And if it may occur twelve days after delivery, there is no reason why it may not fifteen or twenty days after. At the time of the consultation, the patient was so timid, that it required much persuasion on the part of her husband and mother to obtain her consent to have an ordinary vaginal examination. Chiefly on this account it was not deemed advisable to urge an immediate attempt to replace the uterus, but rather to allow the patient a little time to realize her condition, not doubting but she would thereby acquire more fortitude and be in a better condition, both mentally and physically, to undergo so important an operation. I, however, neither saw or heard anything further of the case until Dr. Fisher furnished the report to which these remarks refer.

